# Discursive analysis of news values in headlines posted by the official CCTV News WeChat account

**DOI:** 10.1371/journal.pone.0359636

**Published:** 2026-09-30

**Authors:** Huawei Tan, Ng Miew Luan, Rachel Sing-Ee Tan, Linfei Li

**Affiliations:** 1 School of Literature and Journalism, Chongqing Technology and Business University, Chongqing, China; 2 Faculty of Education and Liberal Arts, INTI International University, Nilai, Malaysia; 3 School of Art and Communication, Chongqing Mobile College, Chongqing, China; Lingnan University, HONG KONG

## Abstract

As social media has become central to news dissemination, how Chinese state media construct news values has remained an underexplored issue. This study analyses how CCTV News uses discursive strategies in WeChat headlines. We used discursive news values analysis (DNVA) to examine 218 top-read headlines from 2014–2024. Five dominant news values emerged: personalization, eliteness, negativity, proximity, and positivity. This study extends the DNVA framework to a non-Western context and offers new insights into the interaction between news language and news values in the context of digital news dissemination.

## Introduction

In the digital age, which is characterized by an information explosion, user attention has become a scarce resource, and social media platforms have rapidly become key venues for news distribution and consumption [1]. However, in a communication environment that is characterized by enhanced user sovereignty, traditional media face unprecedented challenges to their ability to maintain credibility and influence amid fierce competition [2,3]. In particular, it applies to China’s official media that have a mandate for ideological orientation. They should have an adaptive ability for new media communication patterns; maintain balance between their own political purpose and the interests of users when constructing news value [4].

As an important national media organisation of China, the CCTV News official WeChat account quickly gained popularity upon its inception in 2014 and became a typical example for party-affiliated media to transform itself on social media platforms successfully. In terms of the 2024 mainstream media online communication power ranking, according to which CCTV News’ official WeChat account always ranked among the top nationwide WeChat accounts, weekly reader reach exceeded 10 million and weekly likes exceeded one million. Its success has multiple factors: not only because it is rich in authoritative contents and resources but also effective head-line-discourse-adaptations such as strategic-language usage to attract attention; trigger emotion or convey values [5,6]. As the first gate for news release, the headline has become increasingly important in digitalisation. It serves as a critical link connecting media with users or officials to communicate with society at large through emotions [7].

Although a substantial body of research has investigated news values in Western contexts [8], empirical studies on how national media in China construct news values by employing various discourse strategies in the unique political communication environment that characterize this country remain limited [9]. Therefore, a systematic analysis of how state media in China articulate news value discourse on social media platforms is needed.

This study focuses on the CCTV News official WeChat account and, on the basis of the discursive news values analysis (DNVA) theoretical framework, systematically analyses the headlines of the 218 most frequently read articles from 2014–2024. The study aims to reveal how the national media construct news values through discursive strategies. The study focuses on the following two questions:

1. What are the dominant news values that are conveyed through the headlines of the CCTV News official WeChat account?
2. How are these news values constructed in these headlines?

### Literature review

In journalism studies, news values are the fundamental criteria that determine whether an event is newsworthy [10]. News values not only act as aids for journalists in the process of making editorial decisions but also significantly affect the interest of users reading the news, including in terms of their level of engagement [11]. The more the news values dimensions provided in a headline are enriched, the more likely users are to click, comment, and share [12]. The characteristics of resonance, eliteness, personalization, proximity, and negativity in headlines increase the likelihood of high levels of readership [13].

Linguistically, news values are not predetermined phenomena; rather, they are created by journalistic discourse [14,15]. Through the use of lexical selection, syntactic processes and evaluative language, journalists draw the attention of audiences to selected events by identifying and defining them as newsworthy. To uncover the way these discursive strategies create meaning and determine the interface between news content and the user behavior, a DNVA is employed; which is a method that shows the processes of meaning making in news content. The theory by Bednarek and Caple argues that producers of news have preconceptions of what news value entails and that this view finds its way into the headlines under discourse strategies that make users consider some events to be important or worthy of attention. The news discourse, therefore, acts as the third party between the producers and the users, and the language strategy can be viewed as a powerful force that shapes interactions with the audience [16].

Although this theory has been frequently utilised in research related to Western commercial media, so far no thorough investigation exists regarding Chinese mainstream media’s use of discourses for balancing communication power with value guidance when conducting ideological dissemination work under new circumstances. Under a situation where dominant is the communication environment, news headlines should be able to capture user attention and maintain ideological unification through consistent messages [4]. To expand on the use of DNVA within the Chinese context specifically, as this link is so tight between news media and the government in mainland China.

## Methodology

The data source of this study is from the publically available CCTV News official WeChat account, and no individuals’ data were utilised. Based on this context, the consulted data set is composed of 218 articles published from 2014 to 2024, and these have been obtained through purposive sampling. Since this study uses publicly available articles, no identifiable individual participants were involved. The study thus did not involve any personally identifiable information. Only publicly available headline text and aggregated view counts were collected; no user comments, personal data, or private information were accessed or analyzed. The data collection and analysis methods strictly complied with the terms and conditions of the WeChat official account platform.

The year 2014 marks the starting point because it was the earliest year with accessible data after the launch of the CCTV News WeChat account. The year 2024 represents the most recent complete year available at the time the study was conducted.

The CCTV News official WeChat account was chosen as the focus of the analysis for the following reasons. To begin with, WeChat has more than 1.3 billion active users, and the official account system is a key platform on which the national media houses focus on attracting the attention of users and influencing the information. In comparison to Weibo or Tik Tok, WeChat is also associated with a higher degree of information retention and deep reading, alongside considerably more immersive and resonant user experiences [5]. Second, the CCTV News official WeChat account has always topped the media online influence charts, thus indicating that the degree of audience engagement on the WeChat platform is high. The reporting of news by WeChat provides valuable insights for other traditional media facing social media transformation. Lastly, there are relatively few studies on the construction of news values in social media news discourse, and since the group being studied is a popular WeChat account, CCTV News offers a perfect sample to study this phenomenon within the Chinese media context.

This study used the designated publication date feature in WeChat to sample the information on a yearly basis. The WeChat interface displayed up to 20 most-read articles annually (19 in 2018 and 2020; 20 in other years), culminating in a final sample of 218 headlines, as high viewership considered a significant measure of alignment between news values and interest in the audience [17]. Notably, with WeChat displaying view counts only as “100,000+” rather than exact figures, no fixed numerical view-count threshold was imposed.

The coding scheme used for this study is based on the nine dimensions of news values proposed by Bednarek and Caple [18]. These dimensions are a well-established assessment system in news discourse analysis and they may effectively reflect the implicit news value tendencies in headline language [8,16]. The coding rules and corresponding discursive strategies for each news value are detailed in Table 1.

**Table 1:** Discursive strategies pertaining to news values.

News value	Linguistic resources and examples
Consonance	References to stereotypical attributes or preconceptions; Assessments of expectedness/typicality (e.g., “predictable” or “commonly known for”); similarity with the past (e.g., “yet another” or “once again”); Explicit references to general knowledge/traditions, and so on (e.g., “widely recognized”)
Eliteness	Various status markers, including role labels (e.g., “professor” or “CEO”) Representative words indicating status (e.g., “eminent”, “renowned”, or “influential”); recognized names (e.g., “Elon Musk”); descriptions of achievements/fame (e.g., “award-winning” or “bestselling author”)
Impact (having significant effects or consequences)	Assessments of significance (e.g., “groundbreaking”, “pivotal”, or “essential”); representation of actual or potential significant/relevant consequences, including abstract, material, or mental effects (e.g., “that will change everything”, “could lead to a crisis”, or “resulting in widespread disruption”)
Negativity/positivity (negative/positive)	References to negative/positive emotion and attitude (e.g., “devastated”, “praise”, “elated”, or “rejoice”); negative/positive evaluative language (e.g., “awful” or “outstanding”); negative/positive lexis (e.g., “crisis”, “harm”, “loss”, “achievement”, “victory”, or “support”); descriptions of negative (e.g., “unethical”) or positive behavior (e.g., “exceptionally kind”)
Personalization (having a personal/human face)	References to ordinary people, their emotions, and experiences (“Villager: ‘We feel so wronged we could die!’”)
Proximity (being located nearby geographically or culturally)	Explicit references to place or nationality near the target community (“China” or “Hainan”); references to the nation/community via deixes, generic place references, adjectives (“here”, “the nation’s capital”, or “home-grown”)
Superlativeness (high intensity/large scope)	Intensifiers (“severe” or “dramatically”); quantifiers (“thousands” or “huge”); intensified lexis (“panic” or “smash”); comparison (“the largest factory”); repetition (“building after building flattened”); lexis of growth (“a growing list of scaling-up efforts”); only/just/alone/already + time/distance or related lexis (“only hours after”)
Timeliness (recent, ongoing, on the verge of happening, new, current, seasonal)	Temporal references (“today”, “yesterday’s”, “within days”, or “now”); present and present perfect (“it is testing our emergency resources”); implicit time references through lexis (“continues”, “ongoing”, or “have begun to”); reference to current trends, seasonality (“New Year’s Eve”, “Autumn”, or “Twenty-Four Solar Terms”)
Unexpectedness (unexpected)	Evaluations of unexpectedness (“different”, “astonishing”, “strange”), references to surprise/expectations/unusual happenings (“A Money-Counting Machine That ‘Steals Money’!”); comparisons that indicate unusualness (“the first time since 2012”)

It is important to note that all coding was conducted on the original Chinese headlines to preserve linguistic nuances (such as deixis and culturally specific internet slang). The English translations provided in this paper are for illustrative purposes only. Each headline was independently assessed by two coders, who recorded whether each of the ten news values appeared in the headline (1 = presence, 0 = absence). A single headline can reflect multiple news values [16]. To enhance the consistency of the coding process, the research team implemented several measures: first, the two coders familiarized themselves with the coding standards and sample headlines through practice before the formal coding process; second, any discrepancies in coding results were thoroughly discussed and resolved through consensus; and finally, the consistency of the coding was formally tested by calculating the Kappa coefficient [14,19]. Table 2 shows that the Kappa coefficient of the news values ranged between 0.812 and 0.951. According to the established criteria of Burla et al. (2008), these items indicate that the coding approach used in this study is consistent at a higher degree exhibited a high level of reliability [20,21]. There is no participation of people in this study, so ethics review and obtaining informed consent were waived.

**Table 2:** Interrater reliability test for the news value coding of CCTV news headlines.

News Value	Kappa
Consonance	0.834
Eliteness	0.852
Impact	0.862
Timeliness	0.849
Unexpectedness	0.815
Personalization	0.886
Superlativeness	0.833
Proximity	0.831
Negativity	0.951
Positivity	0.830

### Findings

The distribution of news value elements in the headlines of the official CCTV News WeChat account over the past decade is illustrated in Fig 1.

**Fig 1:**
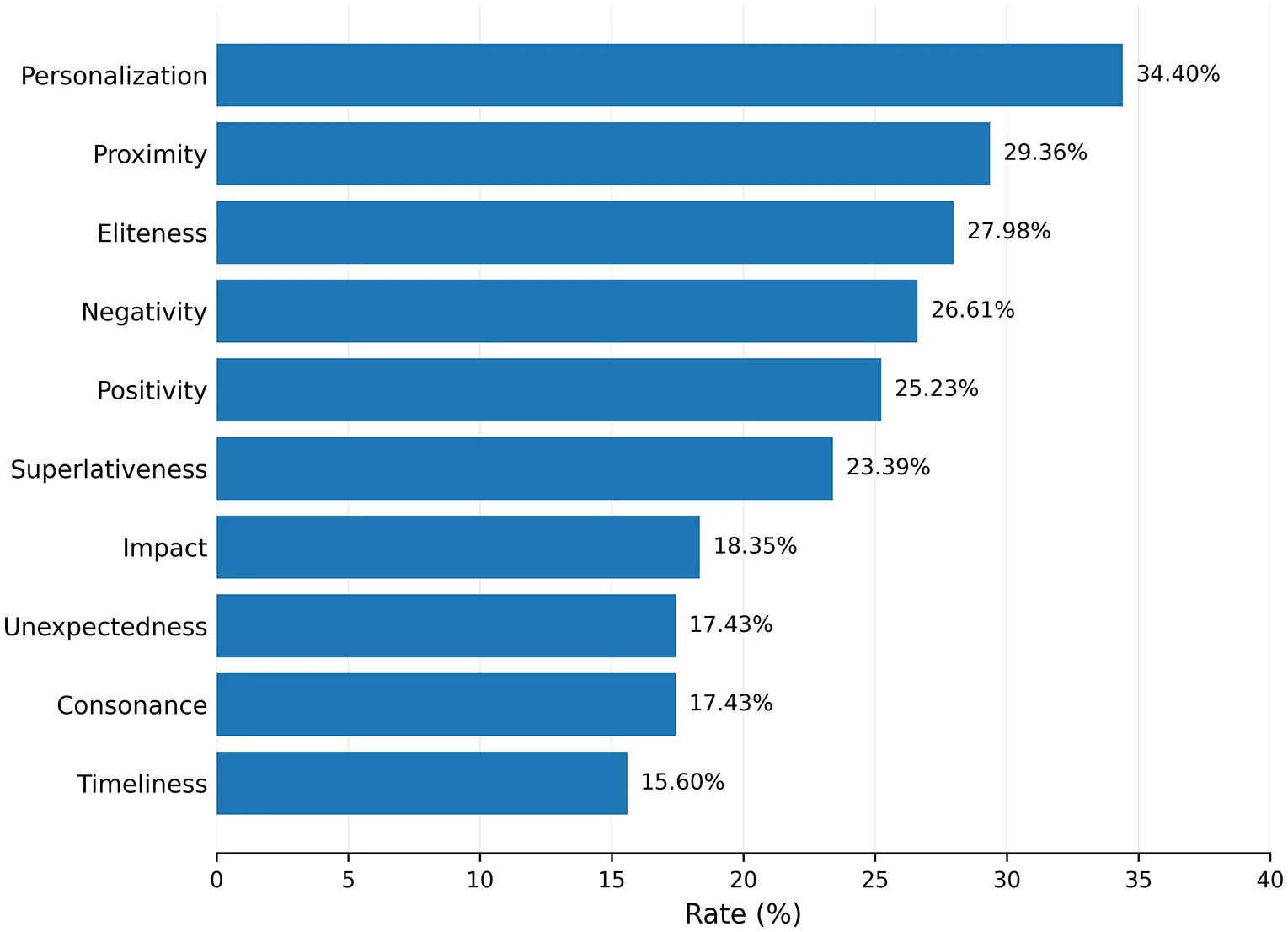
Distribution of the news value elements in CCTV News WeChat Headlines (2014–2024).

In the total number of 218 news headlines studied in this research, personalization was used the most often (n = 75, 34.4%). It was then followed by proximity (n = 64, 29.36%), and eliteness (n = 61, 27.98%) came last. In addition, negativity (n = 58, 26.61%) and positivity (n = 55, 25.23%) also appeared relatively frequently. The news values that appeared less frequently included impact (n = 40, 18.35%), unexpectedness (n = 38, 17.43%), resonance (n = 38, 17.43%), and timeliness (n = 34, 15.06%).

These results indicate that the top three news values each appeared more than 60 times. Furthermore, negativity and positivity—both being highly affective news values—collectively accounted for 113 instances, representing 51.44% of the total. On this basis, the analysis conducted for this study focuses on the top five most frequently occurring news values.

## Discussion

### Personalization as a people-oriented discursive strategy

As a news value, personalization refers to the practice of presenting broader issues in ways that are linked to individual emotions and personal experiences [15]. In news discourse, personalization is realized by highlighting the feelings, experiences, and viewpoints of ordinary people [21].

In the official CCTV News WeChat account, personalized language strategies are frequently observed in news headlines. These strategies include the use of emotive language, emphasis on individual experiences, and the inclusion of voices from ordinary people.

(1) *痛心!男童掉进工地井坑身亡, 谁来为致命的“缺失”负责? (“Heartbreaking! Young Boy Falls into Construction Site Pit and Dies—Who Is Responsible for the Deadly ‘Negligence’?”) (2019-11-12)*
(2) *央视专访奶西村被打少年: 被打后怕见生人 (“CCTV Exclusive Interview with the Beaten Boy from Naixi Village: ‘After Being Beaten, I Am Afraid to See Strangers’”) (2014-06-24)*
(3) *警惕/一个小小的“神器" 就可能把你卡上的信息全部复制!细思极恐 (“Warning: A Tiny ‘Magic Device’ Could Copy All the Data on Your Bank Card! Chilling Upon Reflection” (2017-11-20)*

The headline (1) “Heartbreaking! Young Boy Falls into Construction Site Pit and Dies—Who Is Responsible for the Deadly ‘Negligence’?” employs the highly emotive term “heartbreaking.” Headline (3) uses popular expressions such as “magic device” and “chilling upon reflection,” which are drawn from internet slang and serve to enhance the immediacy and emotional appeal of the news. Headline (2), “After being beaten, I’m afraid to meet anyone”, uses first-person point of view and expresses fear through an immediate sense (“afraid”) in this way to show how the person feels. Generally speaking, these kinds of names are often expressed emotionally in a vivid manner using expressions such as experience, or through colloquial language for expression.

Personalization appears prominently in the headline across the samples through techniques such as emotional expression, direct allusion to individual experience, citation of others’ words or phrases, the inclusion of internet slang, popular expressions etc. Such “people-oriented” linguistic strategies have activated audience interest by using the mechanism of empathy in Chinese political culture to reconcile two aspects – guidance of public sentiment and arousing emotional resonance [4]. Therefore, the mainstream media’s reporting practice can maximally ensure its own direction of people-centred journalism. It is no longer just an engine for increasing user stickiness but also serves to build a cultural identity and political legitimacy [5]. In addition, personalization for the case of newsgathering via social media platforms such as WeChat falls under this general trend of digitised communication adjusting the content according to individual’s interest and social status to improve the effect of disseminating information [22].

### Eliteness as an authoritative discursive strategy

Eliteness as a type of news value refers to individuals or organizations with high visibility and notable achievements [23]. In news discourse, eliteness is realized through certain linguistic strategies, including positional labels, well-known individuals’ names, and citations of recognitions and awards. Eliteness is consistently evident in the news headlines of the official CCTV News WeChat account through various language strategies. These strategies include the use of positional labels (such as “General Secretary” or “Academician”), the mention of celebrity names (such as “Xi Jinping” or “Zhong Nanshan”), and the description of achievements or official events (such as “high-level reception” or “award-winning figures”).

(4) *习近平会见联合国秘书长古特雷斯 (“Xi Jinping Meets with UN Secretary-General Guterres”) (2022-11-16)*
(5) *独家V观/习近平在辽宁考察 (“Exclusive Video/Xi Jinping’s Inspection Tour in Liaoning”) (2018-09-27)*
(6) *习近平访英 英王室将高规格接待 (“Xi Jinping’s Visit to the UK: Royal Family to Provide High-Profile Reception”) (2015-10-16)*
(7) *感动中国2013年度十大人物揭晓 (“The Top Ten Most Inspiring Chinese People of 2013 Announced”) (2014-02-10)*

Headline (4) falls into both the domestically-focused and internationally-oriented categories. Another example of headlines (5) as follows: “Exclusive video | Xi Jinping’s inspection tour in Liaoning”, and has added an exclusive mark. Headlines can also use description of language to express formality or recognition. Headline (6) refers to “High-Profile” in relation to the event. Some headlines highlight either the awards for individual or collective achievements, such as Headline (7): “Top Ten Most Inspirational Chinese of 2013 Have Been Recognised”, which lists recognised individuals.

In the analyzed sample, eliteness was predominantly realised in headlines through a variety of linguistic strategies, including positional titles, well-known names, descriptive terms, and references to recognitions and awards. According to observations by Mañoso-Pacheco in 2020 that there are clear links between elites and determining authorities, representativeness and influencing news-subject constructions. This study also indicates similar relationships among elites [23]. By highlighting the prominent status and achievements of individuals and institutions in headlines, media outlets not only enhance the reach of their reporting but also reinforce the narrative framework of national ideology. Through emphasis on the pivotal position and contributions of outstanding individuals or institutions in the headline to expand coverage and promote shared ideology through this medium.

Furthermore, according to Bednarek and Caple (2017), identity labels have become highly abstract representations of corresponding attributes [18]. In Chinese mainstream media, positional labels such as ‘Xiaomi’, celebrities’ names, positive reviews, and descriptive achievements are generally used to systematically form an authoritative discourse framework. These measures show an institution’s image construction [24], while being closely related to national value orientations and the mainstream social ideology of collectivism and patriotism [9].

Emphasising the news value of eliteness in official Chinese social media news is essential for creating a certain kind of authority and trustworthiness to help realise the government’s plans and restore public confidence [25]. In terms of psychological assistance for the public and promoting a positive attitude towards government policies, at this time, people generally have faith in political leadership as an underlying belief among Chinese society.

### Proximity as a strategy of national identification

Proximity as a type of news value can be defined as geographical, cultural or emotional distance from the target audience for news events [16]. In news writing, a sense of nearness can be achieved in terms of language by explicitly referring to places, using deixis (e.g., “here” or “we”), activating collective memories, etc.

The news headlines frequently carry an official CCTV News WeChat account’s pursuit of closeness by employing various linguistic strategies. Some ways to implement these strategies involve specifically mentioning certain geographical areas (“Kunming”), referencing political or cultural notions (“the Chinese Dream”), and employing deixes to create an atmosphere of immediate presence or common experience.

(8) 昆明火车站严重暴恐案公审 涉故意杀人等罪 *(“Trial of Kunming Railway Station Terrorist Attack: Accused of Intentional Homicide and Other Crimes”) (2014-10-31)*
(9) 两年了, 你真的了解“中国梦*”? (“Two Years On, Do You Really Understand the ‘Chinese Dream’?”) (2014-11-29)*
(10) 习近平行经三省 在辽宁他首先去了这座城市 *(“Xi Jinping Passed Through Three Provinces: In Liaoning, He First Visited This City”) (2018-09-28)*

Headline (8) is located in Kunming, as indicated by the use of the word “Kunming.” Headline (9), “Two Years On—Do You Really Understand the ‘Chinese Dream’?”, combines a sense of national belonging with a direct inquiry to the public. Proximity is also expressed more subtly through language, for example, via vague deixis. In Headline (10), the phrase “this city” functions as a non-specific reference that allows readers to form identification or participate in interpretation. These strategies can also involve second-person pronouns and the activation of shared cultural memories.

In the sample under analysis, proximity is mainly achieved in the headlines by direct geographic names, mentions of collective ideas, deixes, and pronouns that generate an impression of proximity or participation. To ensure that the audience and the content being reported have an emotional attachment, as Bednarek and Caple (2017) note, an aspect of proximity in news communication can be used to create a strong bond between the two. Such strategies facilitate the perceived proximity of the national narrative through the activation of either spatial, cultural, or emotional ties [26].

### Balancing negativity and positivity as a discursive strategy

As news values, negativity and positivity refer to two opposing emotional orientations that form the emotional foundation of news and can significantly influence audience’ attitudes and engagement behaviors [27]. In news discourse, negativity is realized through the presentation of harm, conflict, disasters, or other adverse events, whereas positivity focuses on themes such as success, progress, and happiness [28]. In the official CCTV News WeChat account, language strategies that reflect negativity and positivity are frequently observed in news headlines.

(11) *视频还原苍南城管街头打人真相 (“Video Reconstructs the Truth Behind Cangnan Urban Management Beating Incident”) (2014-04-27)*
(12) *祝福!中国航天! (“Best Wishes! China’s Space Program!”) (2018-04-24)*
(13) *七夕, 领证去呀? (“Qixi Festival, Shall We Get Our Marriage Certificates?”) (2019-08-07)*

For negativity, headlines often include negative vocabulary, critical evaluations, and emotionally charged expressions. Headline (8) uses words such as “Terrorist Attack” and “Homicide” to enhance the sense of tension and immediacy, and also employs emotional expressions such as “heartrending” and “deadly ‘negligence’” to arouse readers’ sense of responsibility towards justice. Headlines can also be evaluative, using words such as “beating” and “truth”; for example, in Headline (11), these expressions convey the public’s perspective on social injustice directly.

Headlines frequently use affirmative expressions, a kind of constructivist language style, and positive recognitions to showcase the national achievements, scientific developments, and cultural warmth. Headline (12) uses terms such as “blessings” and “achievement” to emphasize collective pride and progress. In personal or everyday contexts, Headline (13), “On Qixi, Let’s Go Get a Marriage Certificate?” (August 7, 2019), uses warm and affectionate language combined with holiday imagery, creating a positive and relatable atmosphere.

The negative and positive emotions expressed in news headlines are realized by means such as emotional words, evaluative phrases, mention of conflicts or achievements, urgency and hope combination. This kind of headline combination can maintain an appropriate proportion of social issues to achievements reporting so as to be consistent with many theories’ view that newspapers serve both to warn people and unite them. Positivity is presented as a method for building a supportive and encouraging report style, and in particular, it should take into account the situation of public crisis during the pandemic [9]. In terms of agency, initiative, and results; thus helping to support the governments’ hold on power and narrative about unity through generating feelings of social stability and cooperation among people.

Negative sentiments are typically news features of adverse events such as terrorism or scandals, but these sentiments are carefully managed within Chinese media to conform to political needs and ideological frameworks [25]. An appropriate framework for negative sentiments supports rather than undermines the construction of a “harmonious society.” In Chinese media practice, this balance between negativity and positivity aims to enhance cultural confidence, collective identity, and national pride [9].

## Conclusion

This study conducted an analytic examination of the most-read news headlines published by the official CCTV News WeChat account in 2014–2024 (n = 218), which demonstrated the application of discursive strategies in social media by the national mainstream media in creating news values and improving communication with the audience. The results reveal that the most frequently used types of news values in headlines of CCTV News are personalization, eliteness, proximity, negativity, and positivity. These values, which are conveyed through emotional expression, symbols of authority, geographic and cultural closeness, and emotional tension modulation.

The theoretical contribution of this study lies, on the one hand, in its extension of the discursive news values analysis (DNVA) method to encompass the context of Chinese national media’s social communication, emphasizing a modifiable linguistic space between policy orientation and user attraction. On the other hand, this study our the understanding of news discourse in the digital news era. In terms of practice, these designs provide concrete suggestions on headlines and language use for other media organisations currently undertaking the socialisation process. Inspired by how CCTV News handles tasks to build news value, media can also improve their own effects of cross-media Communication among each other. Specifically, other social media platforms such as Weibo, Douyin and Xiaohongshu can be improved in terms of emotion-evoking power and interaction through personalization enhancement, utilising elitism for brand building, establishing proximity-based connection strategies, adjusting the balance between positive and negative content, etc.

Compared to Western media environments, the news practice in Chinese national media’s social platform is aimed at both public opinion guidance and user acquisition simultaneously. All the reasons are completely different from those of the West, which is that users’ demands guide media market development.

Nevertheless, this research has some deficiencies. The study focuses on the most-read articles from official WeChat accounts. It is possible that these articles have been recommended to users through algorithms based on certain criteria set by the platform. It indicates that many kinds of contents have become widely spread due to various reasons, yet neither users’ inherent interests nor professional selection has been entirely responsible for this. In this way, the research results will not have been fully reflected from an all-encompassing perspective. In the near future, expect to see an increase in various kinds of data sets; therefore, this kind’s generalization ability will strengthen substantially too. On this basis, subsequent studies may use multiple-platform comparison, tracking of users’ behavior, or cross-cultural analysis to explore the communicative effects and mechanisms of different discursive approaches at various levels, such as news language and user behavior in the age of digital communication. They may also examine how these differences affect impact across different platforms and cultural contexts.
