# Supplementary material for: Discursive analysis of news values in headlines posted by the official CCTV News WeChat account
Source: PLoS One. 2026 Sep 30;21(9):e0359636. doi: 10.1371/journal.pone.0359636 (PMC13626345; doi:10.1371/journal.pone.0359636)
Supplement: Supplementary file 2 [file pone.0359636.s002.docx]

**Dear Editor and Reviewers,**

Thank you for your time and the constructive minor revision feedback on our revised manuscript, “Discursive analysis of news values in headlines posted by the official CCTV News WeChat account” (PONE-D-25-52547). We have carefully addressed all three sets of concerns raised by Reviewer 2, as detailed below. All changes in the revised manuscript are highlighted via track changes, and a clean version is also provided as requested.

**Response to Reviewer #2**

**Comment 1 (Engagement effect inferences):** Some surviving claims infer audience engagement effects not supported by the study design, including the Conclusion’s statement that strategies “effectively enhance both the appeal of the news and users’ emotional involvement” and the proximity section’s claim that they “increase the level of audience involvement and engagement.”

**Response:** We sincerely appreciate this important clarification regarding the limits of our data. As requested, we have removed all causal claims linking headline features to unmeasured audience engagement. Specifically:

We deleted the original engagement-related statement from the Conclusion (Page 21) and replaced it with “serve as key discursive devices for constructing appealing and resonant news narratives,” which focuses explicitly on textual construction rather than audience outcomes. We removed the phrase “and thus increase the level of audience involvement and engagement” from the proximity subsection (Page 19).

All remaining references to the functions of news values are now framed strictly as descriptions of discursive practice, or where relevant, explicitly marked as theoretical inferences drawn from prior DNVA scholarship (e.g., Bednarek & Caple, 2017) .

**Comment 2 (Missing methodological transparency):** Two reporting details are missing: (1) the number of headlines drawn per year and the view threshold used for sampling, and (2) whether coding was conducted on Chinese headlines or English translations.

**Response:** We have added both requested details to the Methodology section to improve transparency:

Sampling details: We specify that the WeChat interface displays up to 20 most-read articles annually; 2018 and 2020 yielded 19 articles each, while all other years displayed the full 20, resulting in the final 218-headline corpus. We further clarify that WeChat reports view counts as “100,000+” rather than exact figures, so no fixed numerical view-count threshold was imposed (Page 8).

Coding language: We explicitly note that all coding was conducted on original Chinese headlines to preserve linguistic nuances (e.g., deixis, culturally specific internet slang), and that English translations provided in the manuscript are for illustrative purposes only (Pages 10–11).

**Comment 3 (Reference formatting and consistency):** Please double-check all reference entries to ensure correct details, formatting, and alignment with in-text citations.

**Response:** We have conducted a full audit of all 28 reference entries to ensure compliance with PLOS ONE requirements. We corrected previously incomplete metadata, including:

Adding missing volume/issue numbers and page ranges for 22 entries (e.g., correcting the 2017 Bednarek & Caple citation to the proper journal volume and page range, and adding full pagination for Fiske et al. 2016).

Supplying the full public access URL for the Pew Research Center report (Entry 1) and complete book chapter details (editor, publisher, page range) for Lagerwerf & Govaert (2020, Entry 13).

Standardizing journal title abbreviations and punctuation across all entries to match PLOS ONE formatting guidelines.

We have also verified that every in-text citation maps exactly to an entry in the reference list, with no mismatches or omissions.

We believe these revisions fully address all outstanding concerns, and as noted by the editor, no further external review is required. Thank you again for your careful feedback, which has significantly improved the rigor and transparency of our manuscript. We hope the revised version is now suitable for publication in PLOS ONE.

Sincerely,

Huawei Tan
Corresponding Author
