# Supplementary material for: Discursive analysis of news values in headlines posted by the official CCTV News WeChat account
Source: PLoS One. 2026 Sep 30;21(9):e0359636. doi: 10.1371/journal.pone.0359636 (PMC13626345; doi:10.1371/journal.pone.0359636)
Supplement: Supplementary file 3 [file pone.0359636.s001.docx]

**Dear Editor and Reviewers,**

Thank you for your time and the constructive feedback provided on our manuscript, “Discursive analysis of news values in headlines posted by the official CCTV News WeChat account” (PONE-D-25-52547). We deeply appreciate the reviewers' insightful comments, which have been instrumental in improving the theoretical depth, methodological clarity, and overall quality of our paper.

We have carefully considered all the comments and revised the manuscript accordingly. Below, we provide a point-by-point response detailing how we have addressed each concern. The changes in the revised manuscript have been highlighted for your convenience.

**Response to Reviewer #1**

**Comment 1:** *This manuscript presents a technically sound and well-structured study grounded in established research on news values and discourse analysis (e.g., Bednarek & Caple, 2017). The corpus design and corpus-assisted analytical approach are appropriate and rigorously applied (Baker et al., 2008). The data clearly support the conclusions, and the manuscript is written in clear, standard academic English. Overall, it constitutes a valuable contribution to research on digital news discourse and platform-based journalism. So, I am recommending to cite: [List of 4 citations]*

**Response:** We sincerely thank Reviewer #1 for the positive assessment of our study's structure, methodology, and contribution to the field. We appreciate your valuable recommendations regarding the literature. We have carefully reviewed the suggested citations and incorporated them as follows:

1 **Baker et al. (2008):** We have added this high-quality methodological literature to the Methodology section to further consolidate the foundation of our corpus-assisted analytical approach. *(Please see Page 11, Methodology section)*
2 **Bednarek & Caple (2017):** We have replaced the previous brief citation with a more detailed elaboration of its core arguments to strengthen the theoretical foundation of the DNVA framework in our study. *(Please see Pages 6 and 8, Literature Review and Methodology sections)*
3 **Roman-San-Miguel et al. (2021):** We have cited this literature to extend the discussion on the structural and industrial value of discursive strategies for mainstream media’s platform transformation. *(Please see Page 4, Introduction section)*
4 **Sánchez-Gey Valenzuela et al. (2023):** While we found this paper on reality and fiction in informative discourse to be highly insightful, our study specifically focuses on the discursive construction of news values within the unique political and algorithmic context of Chinese state media (using the DNVA framework). Because the specific focus of our manuscript diverges from the primary scope of this suggested article, we respectfully opted not to include it to maintain the thematic coherence of our reference list. We hope you understand our rationale.

**Response to Reviewer #2**

**Comment 1 (Conceptual Perspective):** *The study would benefit from substantial revisions to enhance its theoretical ambition and analytical depth. From a conceptual standpoint, the contribution remains somewhat limited. While applying the DNVA framework to a non-Western context is valuable, the findings are largely confirmatory rather than transformative. The identification of dominant news values aligns closely with established literature... The manuscript would gain considerably from a more explicit account of how the findings refine, challenge, or expand DNVA, as well as from deeper engagement with scholarship on state communication, discursive legitimacy, and algorithmic governance.*

**Response:**We thank the reviewer for this insightful critique. We agree that the theoretical contribution needed to be more explicitly articulated. In the revised manuscript, we have repositioned the theoretical value of the study from simply verifying the DNVA framework to highlighting its localized adaptation based on Chinese state media practices.
Specifically, we have expanded the Conclusion section to explicitly state:*"The theoretical contribution of this study lies, on the one hand, in its extension of the discursive news values analysis (DNVA) method to encompass the context of Chinese national media’s social communication, emphasizing a modifiable linguistic space between policy orientation and user attraction."* Furthermore, we have incorporated additional discussions in the Discussion section engaging with concepts of state communication and discursive legitimacy, illustrating how news values in this context serve dual purposes of ideological guidance and audience capture. *(Please see Pages 21-22, Conclusion section, and Pages 13-21, Discussion section)*

**Comment 2 (Conceptual Perspective):** *...and the addition of “exclusivity” as a tenth dimension is not developed with sufficient theoretical rigor to constitute a substantive extension of the model.*

**Response:** We fully agree with the reviewer that the addition of "Exclusivity" as a tenth dimension lacked sufficient theoretical justification to constitute a substantive extension of the DNVA framework. To ensure the theoretical rigor of the study, we have completely removed the dimension of "Exclusivity" and all related discussions from the manuscript. The analysis now relies exclusively on the established news value dimensions proposed in the original DNVA framework. We appreciate this valuable suggestion, which has significantly improved the methodological clarity of the paper. *(Please see Pages 8-11, Methodology section, including Table 1 and Table 2)*

**Comment 3 (Analytical Perspective):** *Analytically, the study remains primarily descriptive. The combination of frequency counts and illustrative examples is clear and methodologically sound, but the analysis does not fully unpack the mechanisms linking news values to the political and infrastructural dynamics of the Chinese digital ecosystem.*

**Response:** We appreciate the reviewer’s suggestion to further elaborate on the underlying mechanisms. In response, we have revised the Discussion section to move beyond a primarily descriptive account. In the analysis of key news values—such as personalization and eliteness—we have unpacked how these discursive strategies are connected to the broader political communication context. For instance, we elaborated on how "people-oriented" linguistic strategies (personalization) activate audience interest through empathy mechanisms in Chinese political culture, serving to build cultural identity and political legitimacy rather than just increasing user stickiness. *(Please see Pages 13-21, Discussion section)*

**Comment 4 (Analytical Perspective):** *The reliance on “most-read” articles as a sampling criterion is not sufficiently problematized in light of algorithmic filtering or institutional curation...*

**Response:** We agree that the use of "most-read" articles needs better contextualization. We have added a discussion acknowledging that high view counts are not solely the result of natural user preferences but are inevitably influenced by platform algorithms and institutional curation. We have clarified this in the Limitations section: *"It is possible that these articles have been recommended to users through algorithms based on certain criteria set by the platform. It indicates that many kinds of contents have become widely spread due to various reasons, yet neither users' inherent interests nor professional selection has been entirely responsible for this."* *(Please see Page 8, Methodology section, and Pages 22-23, Conclusion section)*

**Comment 5 (Analytical Perspective):** *...and the assumed relationship between news values and engagement is not empirically interrogated. A more systematic examination of longitudinal patterns and a sharper discussion of platform logics would significantly elevate the analytical contribution.*

**Response:** We acknowledge the reviewer’s valid concern regarding the lack of empirical interrogation of the relationship between news values and audience engagement (which also addresses the concern regarding statistical analysis rigor). In response, we have revised the focus of the study to improve conceptual clarity. Rather than attempting to infer a causal relationship between news values and audience engagement, the revised manuscript now concentrates primarily on the *discursive construction*of news values in headlines. Accordingly, references to "audience engagement" have been substantially reduced throughout the manuscript (including the title and abstract).
Regarding longitudinal patterns and platform logics, while a full longitudinal quantitative analysis falls outside the scope of this qualitative discourse study, we have expanded our discussion on platform logics in the Conclusion, noting how state media practices differ from Western market-driven logics, and we have explicitly listed the need for multi-platform tracking and longitudinal analysis as a key direction for future research.*(Please see Pages 3-5, Abstract and Introduction sections, and Pages 22-23, Conclusion section)*

**Comment 6 (Discussion Section):** *Finally, the discussion section would benefit from moving beyond a structured restatement of findings toward a more synthetic and theoretically ambitious argument. Clarifying the broader implications of the study for understanding how news values operate at the intersection of political authority and platform governance would help position the work more convincingly within ongoing international debates.*

**Response:** Thank you for this valuable guidance. We have revised the Discussion and Conclusion sections to clarify the broader implications of our study. We have incorporated additional analysis showing how these discursive constructions are shaped not only by journalistic considerations but also by the communicative demands of state-affiliated media and the visibility logics of digital platforms. We highlighted that in state-discourse-dominated journalism systems, strategies for constructing news values serve both as emotional appeals and avenues for ideological dissemination, offering a supplementary perspective to Western-centric media research. *(Please see Pages 21-23, Conclusion section)*

We believe that these revisions have thoroughly addressed the reviewers' concerns and significantly elevated the quality of our manuscript. We thank you again for your constructive feedback and hope the revised manuscript is now suitable for publication in PLOS ONE.

Sincerely,

Huawei Tan
Corresponding Author
