# Supplementary material for: Discursive analysis of news values in headlines posted by the official CCTV News WeChat account
Source: PLoS One. 2026 Sep 30;21(9):e0359636. doi: 10.1371/journal.pone.0359636 (PMC13626345; doi:10.1371/journal.pone.0359636)
Supplement: Peer review history

# Peer review: Decision Letter 0

**DOI:** 10.1371/journal.pone.0359636.r001

**Authors:** Chi Kwok

9 Mar 2026

Dear Dr. Tan,

Thank you for submitting your manuscript to PLOS ONE. After careful consideration, we feel that it has merit but does not fully meet PLOS ONE’s publication criteria as it currently stands. Therefore, we invite you to submit a revised version of the manuscript that addresses the points raised during the review process.

Please submit your revised manuscript by Apr 23 2026 11:59PM. If you will need more time than this to complete your revisions, please reply to this message or contact the journal office at plosone@plos.org. When you're ready to submit your revision, log on to https://www.editorialmanager.com/pone/ and select the 'Submissions Needing Revision' folder to locate your manuscript file.

- A letter that responds to each point raised by the academic editor and reviewer(s). You should upload this letter as a separate file labeled 'Response to Reviewers'.
- A marked-up copy of your manuscript that highlights changes made to the original version. You should upload this as a separate file labeled 'Revised Manuscript with Track Changes'.
- An unmarked version of your revised paper without tracked changes. You should upload this as a separate file labeled 'Manuscript'.

If you would like to make changes to your financial disclosure, please include your updated statement in your cover letter. Guidelines for resubmitting your figure files are available below the reviewer comments at the end of this letter.

If applicable, we recommend that you deposit your laboratory protocols in protocols.io to enhance the reproducibility of your results. Protocols.io assigns your protocol its own identifier (DOI) so that it can be cited independently in the future. For instructions see: https://journals.plos.org/plosone/s/submission-guidelines#loc-laboratory-protocols. Additionally, PLOS ONE offers an option for publishing peer-reviewed Lab Protocol articles, which describe protocols hosted on protocols.io. Read more information on sharing protocols at https://plos.org/protocols?utm_medium=editorial-email&utm_source=authorletters&utm_campaign=protocols.

We look forward to receiving your revised manuscript.

Kind regards,

Chi Kwok

Academic Editor

PLOS One

**Journal Requirements:**

1. When submitting your revision, we need you to address these additional requirements.

Please ensure that your manuscript meets PLOS ONE's style requirements, including those for file naming. The PLOS ONE style templates can be found at

https://journals.plos.org/plosone/s/file?id=wjVg/PLOSOne_formatting_sample_main_body.pdf and

https://journals.plos.org/plosone/s/file?id=ba62/PLOSOne_formatting_sample_title_authors_affiliations.pdf

2. In your Methods section, please include additional information about your dataset and ensure that you have included a statement specifying whether the collection and analysis method complied with the terms and conditions for the source of the data.

3. We note that the grant information you provided in the ‘Funding Information’ and ‘Financial Disclosure’ sections do not match.

When you resubmit, please ensure that you provide the correct grant numbers for the awards you received for your study in the ‘Funding Information’ section.

4. Thank you for stating the following financial disclosure:

“5611”

At this time, please address the following queries:

a) Please clarify the sources of funding (financial or material support) for your study. List the grants or organizations that supported your study, including funding received from your institution.

b) State what role the funders took in the study. If the funders had no role in your study, please state: “The funders had no role in study design, data collection and analysis, decision to publish, or preparation of the manuscript.”

c) If any authors received a salary from any of your funders, please state which authors and which funders.

d) If you did not receive any funding for this study, please state: “The authors received no specific funding for this work.”

Please include your amended statements within your cover letter; we will change the online submission form on your behalf.

5. In the online submission form, you indicated that “The data analyzed in this study are publicly available headlines from the “CCTV News” WeChat Official Account (2014–2024). The coded dataset is available from the corresponding author upon reasonable request.”

All PLOS journals now require all data underlying the findings described in their manuscript to be freely available to other researchers, either a. In a public repository, b. Within the manuscript itself, or c. Uploaded as supplementary information.

This policy applies to all data except where public deposition would breach compliance with the protocol approved by your research ethics board. If your data cannot be made publicly available for ethical or legal reasons (e.g., public availability would compromise patient privacy), please explain your reasons on resubmission and your exemption request will be escalated for approval.

6. Your ethics statement should only appear in the Methods section of your manuscript. If your ethics statement is written in any section besides the Methods, please move it to the Methods section and delete it from any other section. Please ensure that your ethics statement is included in your manuscript, as the ethics statement entered into the online submission form will not be published alongside your manuscript.

7. If the reviewer comments include a recommendation to cite specific previously published works, please review and evaluate these publications to determine whether they are relevant and should be cited. There is no requirement to cite these works unless the editor has indicated otherwise.

**Additional Editor Comments:**

The reviewers found your manuscript to be promising, but also suggested some revisions. Therefore, I invite you to respond to the their comments and revise your manuscript.

Reviewers' comments:

**Comments to the Author**

1. Is the manuscript technically sound, and do the data support the conclusions?

Reviewer #1: Yes

Reviewer #2: Partly

**********

2. Has the statistical analysis been performed appropriately and rigorously?

Reviewer #1: Yes

Reviewer #2: No

**********

3. Have the authors made all data underlying the findings in their manuscript fully available?

The PLOS Data policy

Reviewer #1: Yes

Reviewer #2: Yes

**********

4. Is the manuscript presented in an intelligible fashion and written in standard English?

Reviewer #1: Yes

Reviewer #2: Yes

**********

**Reviewer #1:**  This manuscript presents a technically sound and well-structured study grounded in established research on news values and discourse analysis (e.g., Bednarek & Caple, 2017). The corpus design and corpus-assisted analytical approach are appropriate and rigorously applied (Baker et al., 2008). The data clearly support the conclusions, and the manuscript is written in clear, standard academic English.

Overall, it constitutes a valuable contribution to research on digital news discourse and platform-based journalism.

So, I am recommending to cite:

- Baker, P., Gabrielatos, C., KhosraviNik, M., Krzyżanowski, M., McEnery, T., & Wodak, R. (2008). A useful methodological synergy? Combining critical discourse analysis and corpus linguistics. Discourse & Society, 19(3), 273–306.

- Bednarek, M., & Caple, H. (2017). The Discourse of News Values. Oxford: Oxford University Press.

- Roman-San-Miguel, A.; Elías-Zambrano, R., & Paredes Molina, M. (2021). Reality and fiction in informative discourse. Ámbitos. International Journal of Communication, (51), 81–97. https://doi.org/10.12795/Ambitos.2021.i51.06

- Sánchez-Gey Valenzuela, N., Jiménez-Marín, G., & Mancinas-Chávez, R. (2023). The structure of television production in Andalusia as the basis of the regional business fabric. REGIONAL RESEARCH-Journal of REGIONAL RESEARCH, (56), 91-107. https://doi.org/10.38191/iirr-jorr.23.01

**Reviewer #2:**  The manuscript presents a clear methodological design, a well-defined corpus, and strong intercoder reliability, together providing a solid empirical foundation. That said, in its current form, the study would benefit from substantial revisions to enhance its theoretical ambition and analytical depth. From a conceptual standpoint, the contribution remains somewhat limited. While applying the DNVA framework to a non-Western context is valuable, the findings are largely confirmatory rather than transformative. The identification of dominant news values aligns closely with established literature, and the addition of “exclusivity” as a tenth dimension is not developed with sufficient theoretical rigor to constitute a substantive extension of the model. The manuscript would gain considerably from a more explicit account of how the findings refine, challenge, or expand DNVA, as well as from deeper engagement with scholarship on state communication, discursive legitimacy, and algorithmic governance.

Analytically, the study remains primarily descriptive. The combination of frequency counts and illustrative examples is clear and methodologically sound, but the analysis does not fully unpack the mechanisms linking news values to the political and infrastructural dynamics of the Chinese digital ecosystem. The reliance on “most-read” articles as a sampling criterion is not sufficiently problematized in light of algorithmic filtering or institutional curation, and the assumed relationship between news values and engagement is not empirically interrogated. A more systematic examination of longitudinal patterns and a sharper discussion of platform logics would significantly elevate the analytical contribution. Finally, the discussion section would benefit from moving beyond a structured restatement of findings toward a more synthetic and theoretically ambitious argument. Clarifying the broader implications of the study for understanding how news values operate at the intersection of political authority and platform governance would help position the work more convincingly within ongoing international debates.

**********

what does this mean?). If published, this will include your full peer review and any attached files.

If you choose “no”, your identity will remain anonymous but your review may still be made public.

**Do you want your identity to be public for this peer review?**  For information about this choice, including consent withdrawal, please see our Privacy Policy

Reviewer #1: No

Reviewer #2: **Yes:**  Ana Jiménez-Zarco

**********

[NOTE: If reviewer comments were submitted as an attachment file, they will be attached to this email and accessible via the submission site. Please log into your account, locate the manuscript record, and check for the action link "View Attachments". If this link does not appear, there are no attachment files.]

To ensure your figures meet our technical requirements, please review our figure guidelines: https://journals.plos.org/plosone/s/figures

You may also use PLOS’s free figure tool, NAAS, to help you prepare publication quality figures: https://journals.plos.org/plosone/s/figures#loc-tools-for-figure-preparation.

NAAS will assess whether your figures meet our technical requirements by comparing each figure against our figure specifications.

# Author comment: Author response to Decision Letter 1

**DOI:** 10.1371/journal.pone.0359636.r002

2 Apr 2026

Dear Editor and Reviewers,

Thank you for your time and the constructive feedback provided on our manuscript, “Discursive analysis of news values in headlines posted by the official CCTV News WeChat account” (PONE-D-25-52547). We deeply appreciate the reviewers' insightful comments, which have been instrumental in improving the theoretical depth, methodological clarity, and overall quality of our paper.

We have carefully considered all the comments and revised the manuscript accordingly. Below, we provide a point-by-point response detailing how we have addressed each concern. The changes in the revised manuscript have been highlighted for your convenience.

Response to Reviewer #1

Comment 1: This manuscript presents a technically sound and well-structured study grounded in established research on news values and discourse analysis (e.g., Bednarek & Caple, 2017). The corpus design and corpus-assisted analytical approach are appropriate and rigorously applied (Baker et al., 2008). The data clearly support the conclusions, and the manuscript is written in clear, standard academic English. Overall, it constitutes a valuable contribution to research on digital news discourse and platform-based journalism. So, I am recommending to cite: [List of 4 citations]

Response: We sincerely thank Reviewer #1 for the positive assessment of our study's structure, methodology, and contribution to the field. We appreciate your valuable recommendations regarding the literature. We have carefully reviewed the suggested citations and incorporated them as follows:

1Baker et al. (2008): We have added this high-quality methodological literature to the Methodology section to further consolidate the foundation of our corpus-assisted analytical approach. (Please see Page 11, Methodology section)

2Bednarek & Caple (2017): We have replaced the previous brief citation with a more detailed elaboration of its core arguments to strengthen the theoretical foundation of the DNVA framework in our study. (Please see Pages 6 and 8, Literature Review and Methodology sections)

3Sánchez-Gey Valenzuela et al. (2023): We have cited this literature to extend the discussion on the structural and industrial value of discursive strategies for mainstream media’s platform transformation. (Please see Page 11, Methodology section)

4Roman-San-Miguel et al. (2021): While we found this paper on reality and fiction in informative discourse to be highly insightful, our study specifically focuses on the discursive construction of news values within the unique political and algorithmic context of Chinese state media (using the DNVA framework). Because the specific focus of our manuscript diverges from the primary scope of this suggested article, we respectfully opted not to include it to maintain the thematic coherence of our reference list. We hope you understand our rationale.

Response to Reviewer #2

Comment 1 (Conceptual Perspective): The study would benefit from substantial revisions to enhance its theoretical ambition and analytical depth. From a conceptual standpoint, the contribution remains somewhat limited. While applying the DNVA framework to a non-Western context is valuable, the findings are largely confirmatory rather than transformative. The identification of dominant news values aligns closely with established literature... The manuscript would gain considerably from a more explicit account of how the findings refine, challenge, or expand DNVA, as well as from deeper engagement with scholarship on state communication, discursive legitimacy, and algorithmic governance.

Response:We thank the reviewer for this insightful critique. We agree that the theoretical contribution needed to be more explicitly articulated. In the revised manuscript, we have repositioned the theoretical value of the study from simply verifying the DNVA framework to highlighting its localized adaptation based on Chinese state media practices.

Specifically, we have expanded the Conclusion section to explicitly state:"The theoretical contribution of this study lies, on the one hand, in its extension of the discursive news values analysis (DNVA) method to encompass the context of Chinese national media’s social communication, emphasizing a modifiable linguistic space between policy orientation and user attraction." Furthermore, we have incorporated additional discussions in the Discussion section engaging with concepts of state communication and discursive legitimacy, illustrating how news values in this context serve dual purposes of ideological guidance and audience capture. (Please see Pages 21-22, Conclusion section, and Pages 13-21, Discussion section)

Comment 2 (Conceptual Perspective): ...and the addition of “exclusivity” as a tenth dimension is not developed with sufficient theoretical rigor to constitute a substantive extension of the model.

Response: We fully agree with the reviewer that the addition of "Exclusivity" as a tenth dimension lacked sufficient theoretical justification to constitute a substantive extension of the DNVA framework. To ensure the theoretical rigor of the study, we have completely removed the dimension of "Exclusivity" and all related discussions from the manuscript. The analysis now relies exclusively on the established news value dimensions proposed in the original DNVA framework. We appreciate this valuable suggestion, which has significantly improved the methodological clarity of the paper. (Please see Pages 8-11, Methodology section, including Table 1 and Table 2)

Comment 3 (Analytical Perspective): Analytically, the study remains primarily descriptive. The combination of frequency counts and illustrative examples is clear and methodologically sound, but the analysis does not fully unpack the mechanisms linking news values to the political and infrastructural dynamics of the Chinese digital ecosystem.

Response: We appreciate the reviewer’s suggestion to further elaborate on the underlying mechanisms. In response, we have revised the Discussion section to move beyond a primarily descriptive account. In the analysis of key news values—such as personalization and eliteness—we have unpacked how these discursive strategies are connected to the broader political communication context. For instance, we elaborated on how "people-oriented" linguistic strategies (personalization) activate audience interest through empathy mechanisms in Chinese political culture, serving to build cultural identity and political legitimacy rather than just increasing user stickiness. (Please see Pages 13-21, Discussion section)

Comment 4 (Analytical Perspective): The reliance on “most-read” articles as a sampling criterion is not sufficiently problematized in light of algorithmic filtering or institutional curation...

Response: We agree that the use of "most-read" articles needs better contextualization. We have added a discussion acknowledging that high view counts are not solely the result of natural user preferences but are inevitably influenced by platform algorithms and institutional curation. We have clarified this in the Limitations section: "It is possible that these articles have been recommended to users through algorithms based on certain criteria set by the platform. It indicates that many kinds of contents have become widely spread due to various reasons, yet neither users' inherent interests nor professional selection has been entirely responsible for this." (Please see Page 8, Methodology section, and Pages 22-23, Conclusion section)

Comment 5 (Analytical Perspective): ...and the assumed relationship between news values and engagement is not empirically interrogated. A more systematic examination of longitudinal patterns and a sharper discussion of platform logics would significantly elevate the analytical contribution.

Response: We acknowledge the reviewer’s valid concern regarding the lack of empirical interrogation of the relationship between news values and audience engagement (which also addresses the concern regarding statistical analysis rigor). In response, we have revised the focus of the study to improve conceptual clarity. Rather than attempting to infer a causal relationship between news values and audience engagement, the revised manuscript now concentrates primarily on the discursive constructionof news values in headlines. Accordingly, references to "audience engagement" have been substantially reduced throughout the manuscript (including the title and abstract).

Regarding longitudinal patterns and platform logics, while a full longitudinal quantitative analysis falls outside the scope of this qualitative discourse study, we have expanded our discussion on platform logics in the Conclusion, noting how state media practices differ from Western market-driven logics, and we have explicitly listed the need for multi-platform tracking and longitudinal analysis as a key direction for future research.(Please see Pages 3-5, Abstract and Introduction sections, and Pages 22-23, Conclusion section)

Comment 6 (Discussion Section): Finally, the discussion section would benefit from moving beyond a structured restatement of findings toward a more synthetic and theoretically ambitious argument. Clarifying the broader implications of the study for understanding how news values operate at the intersection of political authority and platform governance would help position the work more convincingly within ongoing international debates.

Response: Thank you for this valuable guidance. We have revised the Discussion and Conclusion sections to clarify the broader implications of our study. We have incorporated additional analysis showing how these discursive constructions are shaped not only by journalistic considerations but also by the communicative demands of state-affiliated media and the visibility logics of digital platforms. We highlighted that in state-discourse-dominated journalism systems, strategies for constructing news values serve both as emotional appeals and avenues for ideological dissemination, offering a supplementary perspective to Western-centric media research. (Please see Pages 21-23, Conclusion section)

We believe that these revisions have thoroughly addressed the reviewers' concerns and significantly elevated the quality of our manuscript. We thank you again for your constructive feedback and hope the revised manuscript is now suitable for publication in PLOS ONE.

Sincerely,

Huawei Tan

Corresponding Author

Attachment Submitted filename: Response to Reviewer.docx

# Editor report: Decision Letter 1

**DOI:** 10.1371/journal.pone.0359636.r003

**Authors:** Chi Kwok

6 Aug 2026

Dear Dr. Tan,

Thank you for your patience while this manuscript was under consideration, and thank you for the care you have taken in answering the reviewers. The revision addresses the substance of what was raised in the previous round. I am pleased to say the paper can move toward publication after one further round of minor revision.

=========================================

Reviewer 2 asked you to stop inferring an engagement effect the data cannot support, and you agreed. Some of those claims survive. The Conclusion still states that the strategies identified “effectively enhance both the appeal of the news and users’ emotional involvement,” and the proximity section still states that they “increase the level of audience involvement and engagement.” Since the design does not measure involvement, please rewrite these as claims about how headlines are constructed, or mark them plainly as inferences carried over from earlier work.

Two details of reporting are missing. The Methods do not say how many headlines were drawn per year or what the view threshold was, and they do not say whether coding was carried out on the Chinese headlines or on English translations. Both are matters of transparency and can be settled in a sentence each.

Please double-check all the reference entries to ensure that the details and formatting are correct, and that the in-text citations are consistent with the reference list.

I do not expect these changes to require further external review. Please return the revision within 30 days, together with a point-by-point response and a tracked-changes file that matches the clean manuscript.

================================

Please submit your revised manuscript by Sep 20 2026 11:59PM. If you will need more time than this to complete your revisions, please reply to this message or contact the journal office at plosone@plos.org. When you're ready to submit your revision, log on to https://www.editorialmanager.com/pone/ and select the 'Submissions Needing Revision' folder to locate your manuscript file.`

Please include the following items when submitting your revised manuscript:

- A rebuttal letter that responds to each point raised by the academic editor and reviewer(s). You should upload this letter as a separate file labeled 'Response to Reviewers'.
- A marked-up copy of your manuscript that highlights changes made to the original version. You should upload this as a separate file labeled 'Revised Manuscript with Track Changes'.
- An unmarked version of your revised paper without tracked changes. You should upload this as a separate file labeled 'Manuscript'.

If you would like to make changes to your financial disclosure, please include your updated statement in your cover letter. Guidelines for resubmitting your figure files are available below the reviewer comments at the end of this letter.

If applicable, we recommend that you deposit your laboratory protocols in protocols.io to enhance the reproducibility of your results. Protocols.io assigns your protocol its own identifier (DOI) so that it can be cited independently in the future. For instructions see: https://journals.plos.org/plosone/s/submission-guidelines#loc-laboratory-protocols. Additionally, PLOS ONE offers an option for publishing peer-reviewed Lab Protocol articles, which describe protocols hosted on protocols.io. Read more information on sharing protocols at https://plos.org/protocols?utm_medium=editorial-email&utm_source=authorletters&utm_campaign=protocols.

As the corresponding author, your ORCID iD is verified in the submission system and will appear in the published article. PLOS supports the use of ORCID, and we encourage all coauthors to register for an ORCID iD and use it as well. Please encourage your coauthors to verify their ORCID iD within the submission system before final acceptance, as unverified ORCID iDs will not appear in the published article. Only the individual author can complete the verification step; PLOS staff cannot verify ORCID iDs on behalf of authors.

We look forward to receiving your revised manuscript.

Kind regards,

Chi Kwok

Academic Editor

PLOS One

Journal Requirements:

If the reviewer comments include a recommendation to cite specific previously published works, please review and evaluate these publications to determine whether they are relevant and should be cited. There is no requirement to cite these works unless the editor has indicated otherwise.

Please review your reference list to ensure that it is complete and correct. If you have cited papers that have been retracted, please include the rationale for doing so in the manuscript text, or remove these references and replace them with relevant current references. Any changes to the reference list should be mentioned in the rebuttal letter that accompanies your revised manuscript. If you need to cite a retracted article, indicate the article’s retracted status in the References list and also include a citation and full reference for the retraction notice.

[Note: HTML markup is below. Please do not edit.]

[NOTE: If reviewer comments were submitted as an attachment file, they will be attached to this email and accessible via the submission site. Please log into your account, locate the manuscript record, and check for the action link "View Attachments". If this link does not appear, there are no attachment files.]

To ensure your figures meet our technical requirements, please review our figure guidelines: https://journals.plos.org/plosone/s/figures

You may also use PLOS’s free figure tool, NAAS, to help you prepare publication quality figures: https://journals.plos.org/plosone/s/figures#loc-tools-for-figure-preparation.

NAAS will assess whether your figures meet our technical requirements by comparing each figure against our figure specifications.

# Author comment: Author response to Decision Letter 2

**DOI:** 10.1371/journal.pone.0359636.r004

11 Aug 2026

Dear Editor and Reviewers,

Thank you for your time and the constructive minor revision feedback on our revised manuscript, “Discursive analysis of news values in headlines posted by the official CCTV News WeChat account” (PONE-D-25-52547). We have carefully addressed all three sets of concerns raised by Reviewer 2, as detailed below. All changes in the revised manuscript are highlighted via track changes, and a clean version is also provided as requested.

Response to Reviewer #2

Comment 1 (Engagement effect inferences): Some surviving claims infer audience engagement effects not supported by the study design, including the Conclusion’s statement that strategies “effectively enhance both the appeal of the news and users’ emotional involvement” and the proximity section’s claim that they “increase the level of audience involvement and engagement.”

Response: We sincerely appreciate this important clarification regarding the limits of our data. As requested, we have removed all causal claims linking headline features to unmeasured audience engagement. Specifically:

We deleted the original engagement-related statement from the Conclusion (Page 21) and replaced it with “serve as key discursive devices for constructing appealing and resonant news narratives,” which focuses explicitly on textual construction rather than audience outcomes. We removed the phrase “and thus increase the level of audience involvement and engagement” from the proximity subsection (Page 19).

All remaining references to the functions of news values are now framed strictly as descriptions of discursive practice, or where relevant, explicitly marked as theoretical inferences drawn from prior DNVA scholarship (e.g., Bednarek & Caple, 2017) .

Comment 2 (Missing methodological transparency): Two reporting details are missing: (1) the number of headlines drawn per year and the view threshold used for sampling, and (2) whether coding was conducted on Chinese headlines or English translations.

Response: We have added both requested details to the Methodology section to improve transparency:

Sampling details: We specify that the WeChat interface displays up to 20 most-read articles annually; 2018 and 2020 yielded 19 articles each, while all other years displayed the full 20, resulting in the final 218-headline corpus. We further clarify that WeChat reports view counts as “100,000+” rather than exact figures, so no fixed numerical view-count threshold was imposed (Page 8).

Coding language: We explicitly note that all coding was conducted on original Chinese headlines to preserve linguistic nuances (e.g., deixis, culturally specific internet slang), and that English translations provided in the manuscript are for illustrative purposes only (Pages 10–11).

Comment 3 (Reference formatting and consistency): Please double-check all reference entries to ensure correct details, formatting, and alignment with in-text citations.

Response: We have conducted a full audit of all 28 reference entries to ensure compliance with PLOS ONE requirements. We corrected previously incomplete metadata, including:

Adding missing volume/issue numbers and page ranges for 22 entries (e.g., correcting the 2017 Bednarek & Caple citation to the proper journal volume and page range, and adding full pagination for Fiske et al. 2016).

Supplying the full public access URL for the Pew Research Center report (Entry 1) and complete book chapter details (editor, publisher, page range) for Lagerwerf & Govaert (2020, Entry 13).

Standardizing journal title abbreviations and punctuation across all entries to match PLOS ONE formatting guidelines.

We have also verified that every in-text citation maps exactly to an entry in the reference list, with no mismatches or omissions.

We believe these revisions fully address all outstanding concerns, and as noted by the editor, no further external review is required. Thank you again for your careful feedback, which has significantly improved the rigor and transparency of our manuscript. We hope the revised version is now suitable for publication in PLOS ONE.

Sincerely,

Huawei Tan

Corresponding Author

Attachment Submitted filename: Response to Reviewer8.11.docx

# Editor report: Decision Letter 2

**DOI:** 10.1371/journal.pone.0359636.r005

**Authors:** Chi Kwok

16 Sep 2026

Discursive analysis of news values in headlines posted by the official CCTV News WeChat account

PONE-D-25-52547R2

Dear Dr. Tan,

We’re pleased to inform you that your manuscript has been judged scientifically suitable for publication and will be formally accepted for publication once it meets all outstanding technical requirements.

Within one week, you’ll receive an e-mail detailing the required amendments. When these have been addressed, you’ll receive a formal acceptance letter and your manuscript will be scheduled for publication.

An invoice will be generated when your article is formally accepted. Please note, if your institution has a publishing partnership with PLOS and your article meets the relevant criteria, all or part of your publication costs will be covered. Please make sure your user information is up-to-date by logging into Editorial Manager at Editorial Manager® and clicking the ‘Update My Information' link at the top of the page. For questions related to billing, please contact billing support.

If your institution or institutions have a press office, please notify them about your upcoming paper to help maximize its impact. If they’ll be preparing press materials, please inform our press team as soon as possible -- no later than 48 hours after receiving the formal acceptance. Your manuscript will remain under strict press embargo until 2 pm Eastern Time on the date of publication. For more information, please contact onepress@plos.org.

Kind regards,

Chi Kwok

Academic Editor

PLOS One

Additional Editor Comments (optional):

Reviewers' comments:

# Editor report: Acceptance letter

**DOI:** 10.1371/journal.pone.0359636.r006

**Authors:** Chi Kwok

PONE-D-25-52547R2

PLOS One

Dear Dr. Tan,

I'm pleased to inform you that your manuscript has been deemed suitable for publication in PLOS One. Congratulations! Your manuscript is now being handed over to our production team.

At this stage, our production department will prepare your paper for publication. This includes ensuring the following:

* All references, tables, and figures are properly cited

* All relevant supporting information is included in the manuscript submission,

* There are no issues that prevent the paper from being properly typeset

You will receive further instructions from the production team, including instructions on how to review your proof when it is ready. Please keep in mind that we are working through a large volume of accepted articles, so please give us a few days to review your paper and let you know the next and final steps.

Lastly, if your institution or institutions have a press office, please let them know about your upcoming paper now to help maximize its impact. If they'll be preparing press materials, please inform our press team within the next 48 hours. Your manuscript will remain under strict press embargo until 2 pm Eastern Time on the date of publication. For more information, please contact onepress@plos.org.

You will receive an invoice from PLOS for your publication fee after your manuscript has reached the completed accept phase. If you receive an email requesting payment before acceptance or for any other service, this may be a phishing scheme. Learn how to identify phishing emails and protect your accounts at https://explore.plos.org/phishing.

If we can help with anything else, please email us at customercare@plos.org.

Thank you for submitting your work to PLOS One and supporting open access.

Kind regards,

PLOS One Editorial Office Staff

on behalf of

Dr. Chi Kwok

Academic Editor

PLOS One
